# Supplementary material for: JAC4 Inhibits EGFR-Driven Lung Adenocarcinoma Growth and Metastasis through CTBP1-Mediated JWA/AMPK/NEDD4L/EGFR Axis
Source: Int J Mol Sci. 2023 May 15;24(10):8794. doi: 10.3390/ijms24108794 (PMC10218353; doi:10.3390/ijms24108794)
Supplement: Supplementary file 1 [file ijms-24-08794-s001.zip › ijms-2231325-supplementary.pdf]

JAC4 Inhibits EGFR-Driven Lung Adenocarcinoma Growth and Metastasis through CTBP1-Mediated JWA/AMPK/NEDD4L/EGFR Axis

Authors:

Kun Ding [1,2,3], Xuqian Jiang [1,2,3], Zhangding Wang [1,2,3], Lu Zou [1,2,3],

Jiahua Cui [1,2,3], Xiong Li [1,2,3], Chuanjun Shu [4], Aiping Li [1,2,3]

and Jianwei Zhou [1,2,3,*]

[1] Department of Molecular Cell Biology & Toxicology, Center for Global Health, School of Public Health, Nanjing Medical University, Nanjing 211166, China

[2] Key Laboratory of Modern Toxicology of Ministry of Education, School of Public Health, Nanjing Medical University, Nanjing 211166, China

[3] Jiangsu Key Lab of Cancer Biomarkers, Prevention and Treatment, Collaborative Innovation Center for Cancer Medicine, Nanjing Medical University, Nanjing 211166, China

[4] Department of Bioinformatics, School of Biomedical Engineering and Informatics, Nanjing Medical University, Nanjing 211166, China

* Correspondence: jwzhou@njmu.edu.cn; Tel./Fax: +86-25-8686-8421

This file includes:

Supplementary methods

Supplementary Figure S1 to S9

Supplementary Tables S1 to S3

Supplementary methods

Public Database Analysis

TCGA datasets (https://xenabrowser.net) were downloaded for analysis of JWA expression or NEDD4L expression in lung cancer tissues and normal lung tissues. Two independent cohorts of lung cancer and corresponding normal lung data (GSE19188 and GSE19804) were downloaded from the Gene Expression Omnibus (GEO) database. GSE19188 has 91 tumor and 65 adjacent normal lung tissue samples; GSE19804 contains 60 pairs of lung cancer samples and adjacent nontumor tissues, respectively. GSE72094 contains the clinical follow-up data of lung cancers, these data were used for the analysis of the correlation between JWA or NEDD4L expression levels and prognosis of lung cancer patients. Lung cancer proteomics data were downloaded from The National Cancer Institute’s Clinical Proteomic Tumor Analysis Consortium (CPTAC, https://proteomics.cancer.gov/) for analysis the protein expression of JWA or NEDD4L in LUAD and LUSC. Besides, The Human Protein Atlas (https://www.proteinatlas.org) was used to verify the JWA or NEDD4L protein expression level in lung cancer. Protein expression of JWA across normal human tissues were analyzed from the Human Proteome Map (www.humanproteomemap.org).Correlation analysis of gene expression in lung patients or lung cancer cell lines was obtained from the TCGA and the Cancer Cell Line Encyclopedia (CCLE) database (https://portals.broadinstitute.org).

Synthesis of biotin-JAC4

The specific synthetic route was shown in the following diagram.

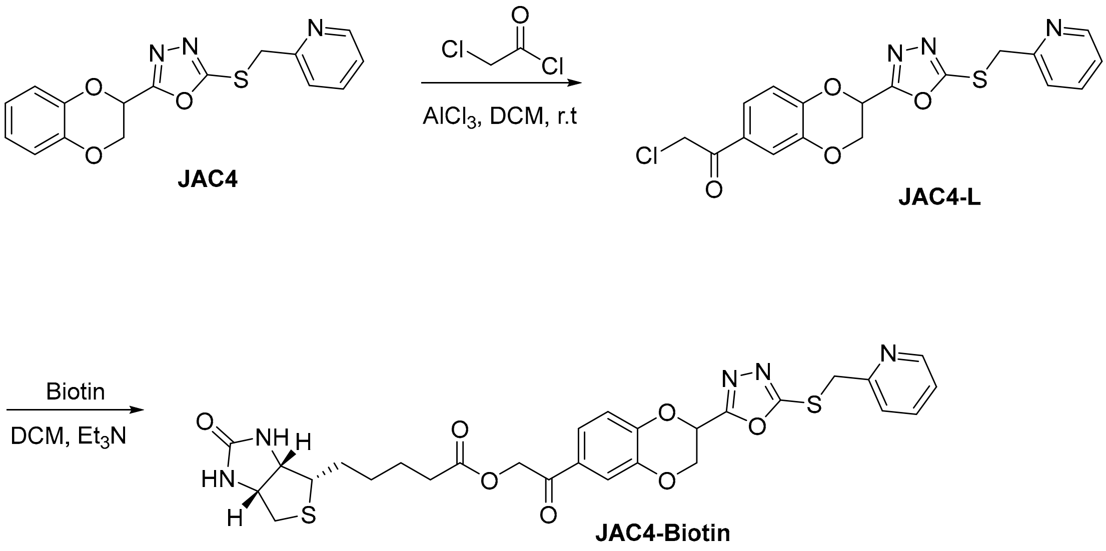
JAC4
AlCl$_3$, DCM, r.t
JAC4-L
Biotin
DCM, Et$_3$N
JAC4-Biotin

Supplementary Figures

Figure S1

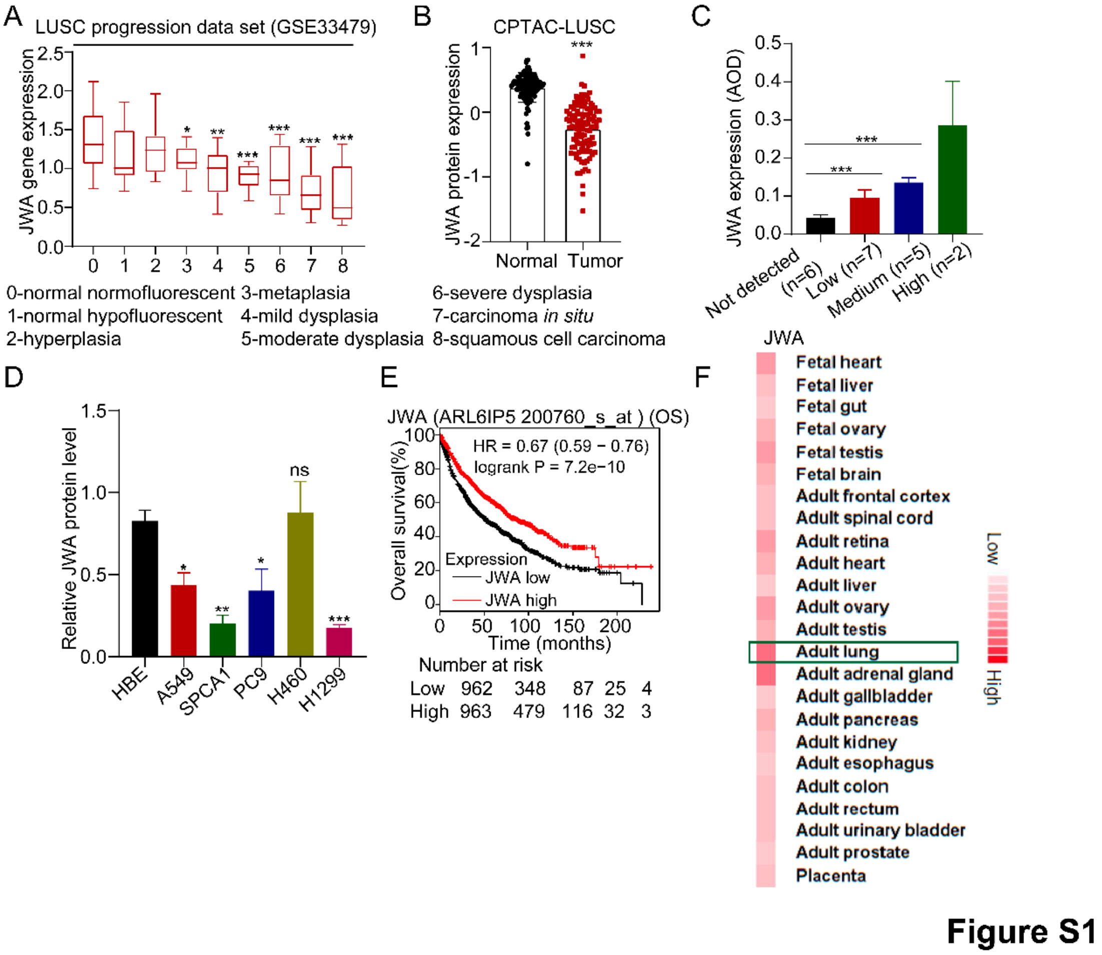

Figure S1

Supplemental Figure S1. JWA expression is downregulated in lung cancer

A. JWA expression in different developmental stages of LUSC tumorigenesis. **B.** Protein expression of JWA in LUSC proteomes (N=T=110) from CPTAC. **C.** JWA Staining was quantified by average optical density (AOD) via Image J. **D.** Quantitative analysis of protein levels in cell lines, n=3 independent experiments. **E.** Kaplan-Meier survival curves of overall survival (OS) based on JWA expression from the online bioinformatics tool Kaplan-Meier Plotter. **F.** Protein expression of JWA in normal human tissues assessed by mass spectrometry from the Human Proteome MAP (www.humanproteomemap.org). Data information: data are presented as mean±SD.

***P <0.001.

Figure S2

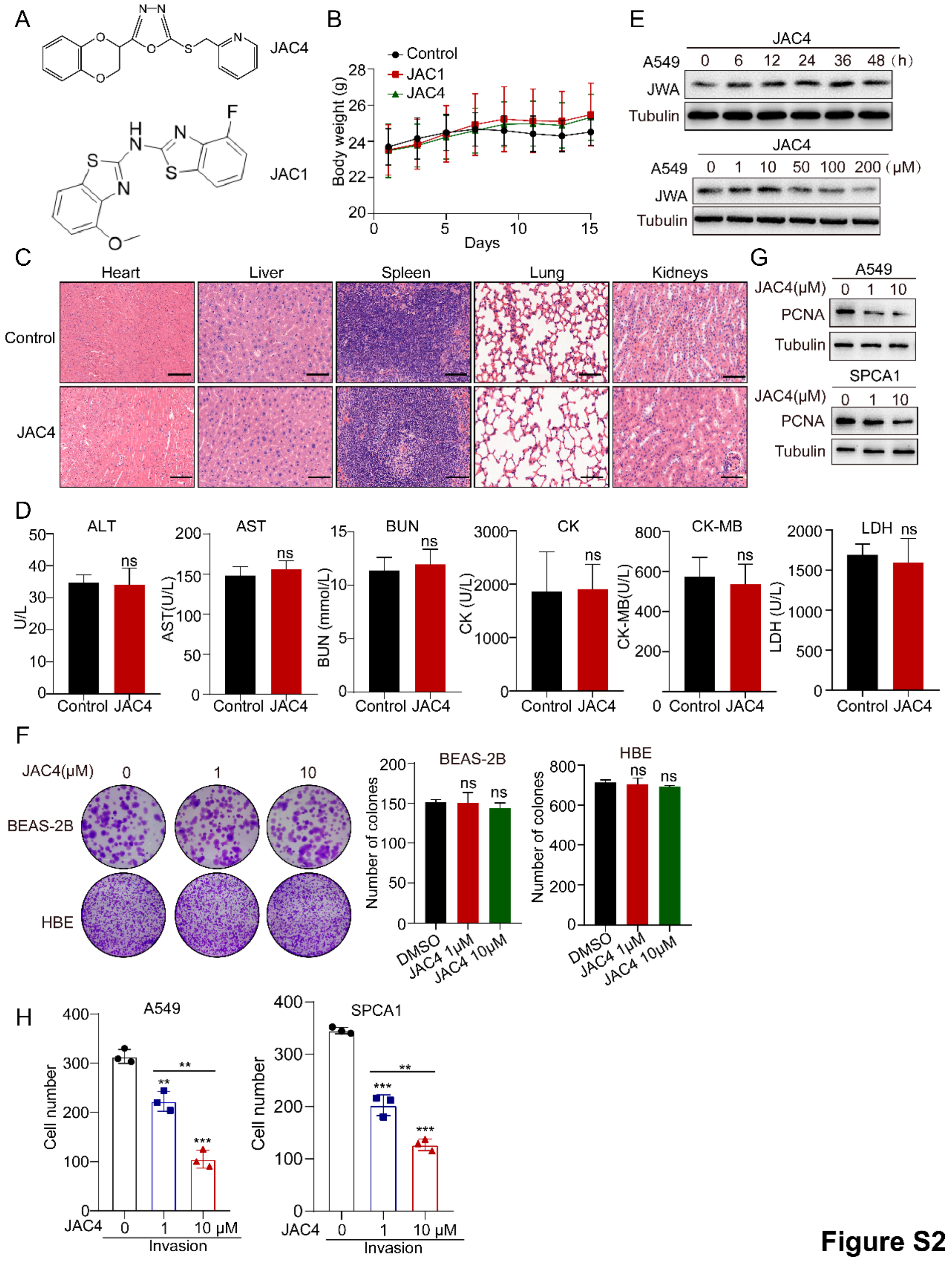

Supplemental Figure S2. JAC4 treatment reveals no signs of toxicity

A Structure of JWA agonist JAC1 and JAC4. **B** Curves of body weight from different treatment groups (Control, JAC4: 100 mg/kg, JAC1 :100 mg/kg) (n=7). **C**

Representative images of various organs by hematoxylin and eosin staining (H&E). Scale bars=100 μm. **D** The main blood biochemical indexes of mice, n=3. **E** Detection of JWA protein expression in A549 cells treated with JAC4 at different hours and concentrations by western blot. **F** Colony formation assays showed no obvious toxic effects on normal lung epithelial cells (BEAS-2B and HBE), n=3 independent experiments. **G** Immunoblot analysis of PCNA (marker of cell proliferation) protein levels in A549 and SPCA1 cells treated different concentrations of JAC4. **H** Quantitative analysis of the effect of JAC4 on the invasion assays of A549 and SPCA1 cells, n=3. Data information: data are presented as mean±SD. **P* <0.05, ***P* <0.01, ****P* <0.001. ns: not significant.

Figure S3

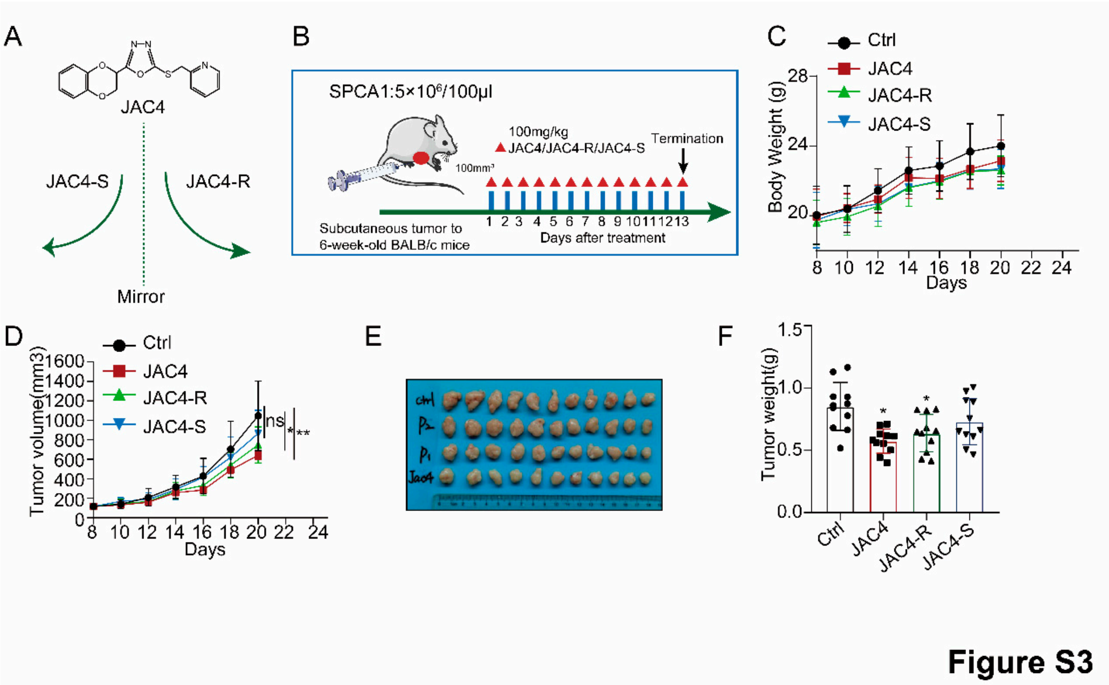

Supplemental Figure S3. Effect of different chirality of JAC4 on tumor growth *in vivo*

A Diagram of different chirality of JAC4. **B** Schematic representation of tumor-bearing

models from different chiral molecular treatments. **C, D** Body weight (C) and tumor volumes (D) in the vehicle, JAC4, JAC4-R, JAC4-S treated groups (n=11). **E, F** Harvested tumor tissues (E) and tumor weight (F) in groups from vehicle, JAC4, JAC4-R and JAC4-S treated, n=11. Data information: data are presented as mean±SD. **P* <0.05, ***P* <0.01. ns: not significant.

Figure S4

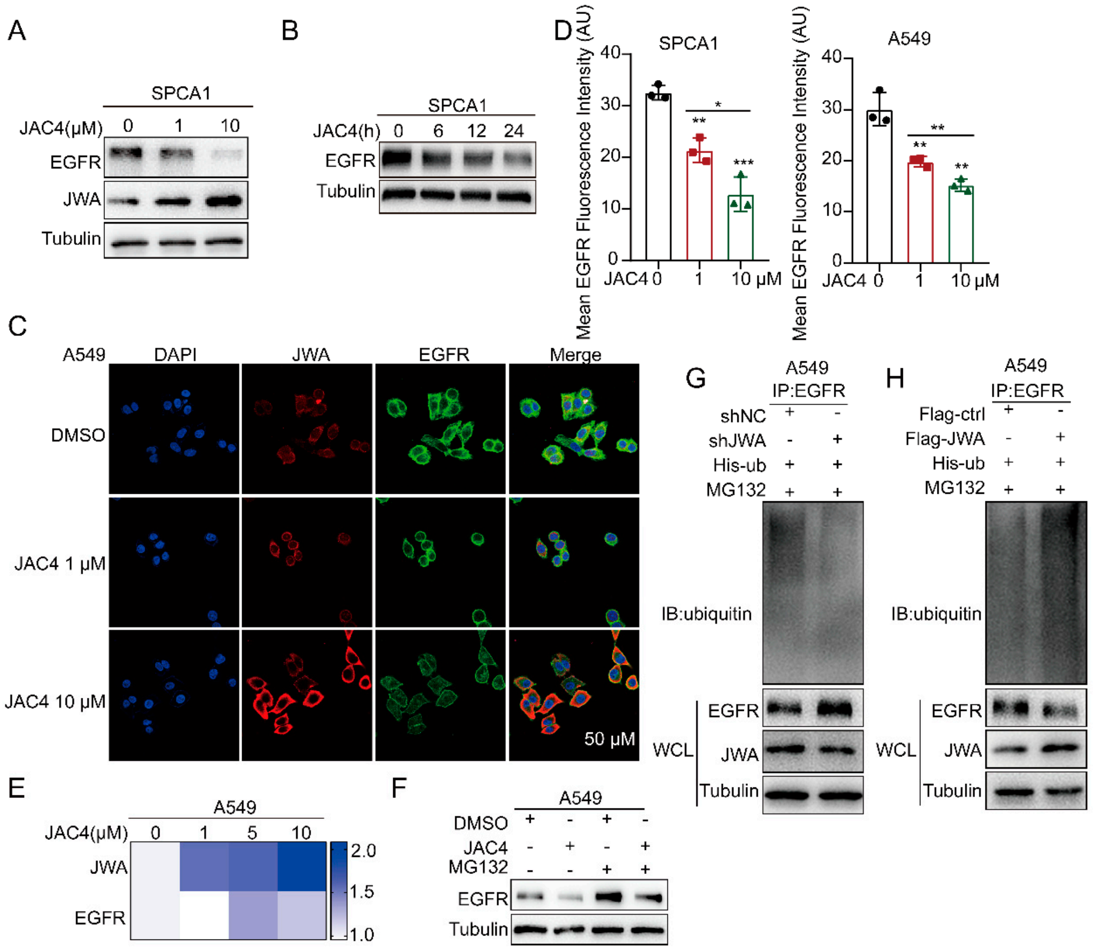

Figure S4

Supplemental Figure S4. JAC4-mediated degradation of EGFR by the ubiquitination pathway

A, B Detection of JWA protein expression in A549 cells treated with JAC4 at different hours (B) and concentrations (A) by western blot. **C, D** Immunofluorescence (IF)

analysis of JWA and EGFR expression in A549 treated with DMSO or JAC4 (n=3). Scale bars=50 μm. **E** Assessment of JWA and EGFR in A549 cells from treatment with different concentrations of JAC4, detected by qRT-PCR. **F** Treatment with protease inhibitors (MG132, 10 μM) attenuated JAC4-mediated EGFR degradation. A549 cells were treated with DMSO or JAC4 (10 μM) for 24 h, then incubation with or without MG132 for 6 hours. **G, H** In A549 cells, high expression of JWA promoted EGFR ubiquitination, however, low expression of JWA inhibited EGFR ubiquitination. Data information: data are presented as mean±SD. ns: not significant.

Figure S5

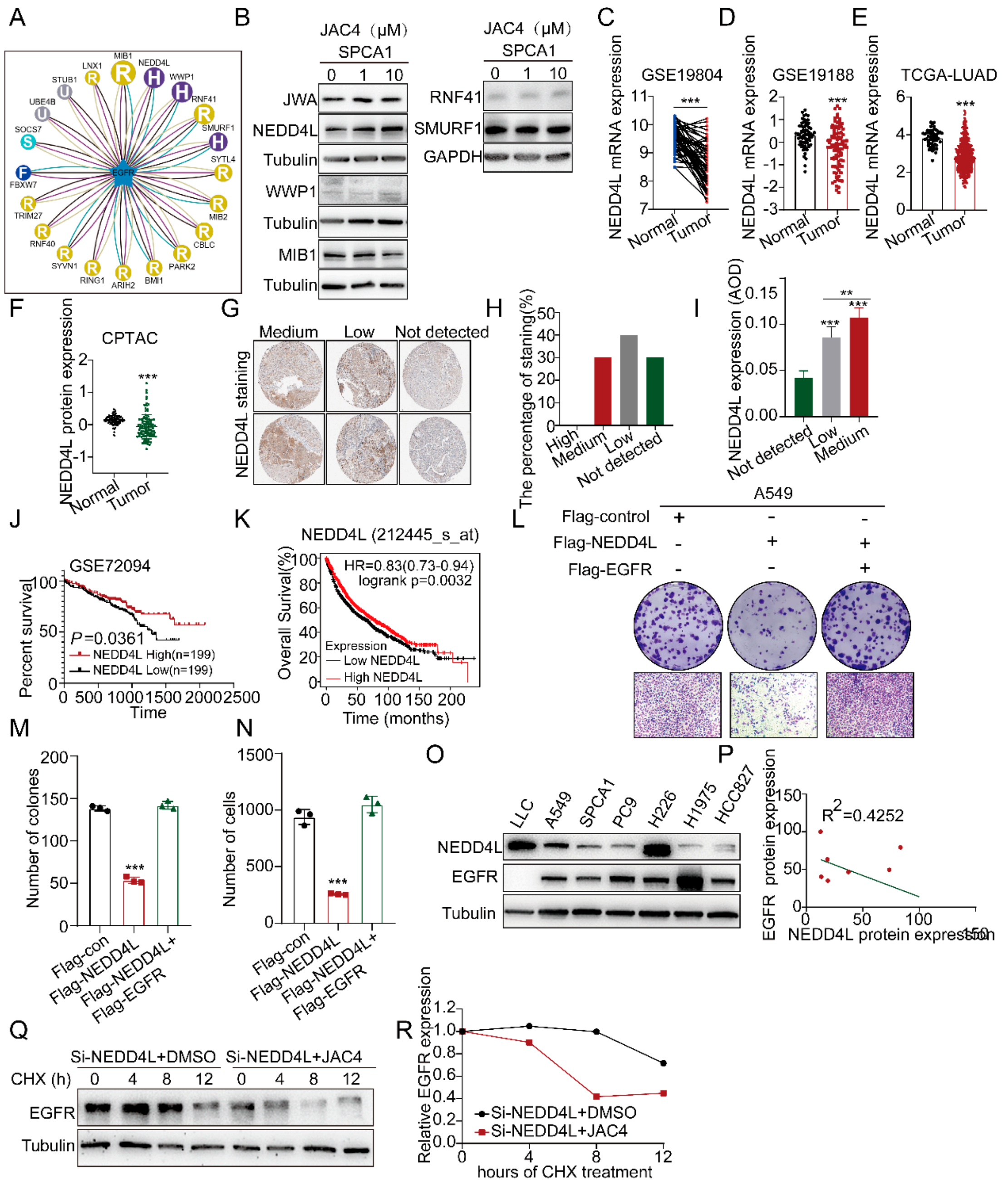

Figure S5

Supplemental Figure S5. NEDD4L inhibits lung cancer proliferation and migration through ubiquitination of EGFR

A The potential E3 ubiquitin ligase of EGFR using online bioinformatics UbiBrowser evidence mode. **B** IB analysis of the top 5 predicted ubiquitin ligase of EGFR. **C, D, E** Relative NEDD4L mRNA expression in lung cancer tissues (GSE19804, GSE19188

and TCGA data set) compared to corresponding control lung tissues. **F** Protein expression of NEDD4L in LUAD proteome (N=101, T=110) from CPTAC. **G, H** Representative IHC images and analysis on tissue microarray probed with anti-NEDD4L antibody in Human Protein Atlas. **I** Quantitative analysis of IHC staining by AOD. **J** Kaplan-Meier survival curves of OS based on NEDD4L expression from GSE72094 dataset. **K** Kaplan-Meier survival curves of overall survival (OS) based on NEDD4L expression in lung cancer from the online bioinformatics tool Kaplan-Meier Plotter. **L, M, N** The colony formation and migration of A549 cells transfected with Flag-control, Flag-NEDD4L or co-transfected with Flag-NEDD4L and Flag-EGFR plasmids by colony formation assay and Transwell assay, n=3 independent experiments. Scale bars=50 μm. **O, P** Negative correlation between NEDD4L and EGFR protein levels in NSCLC cell lines by western blot. **Q, R** A549 cells were transfected with siNEDD4L to inhibit NEDD4L expression, with or without JAC4 intervention, and the protein stability of EGFR in cells was measured by the "CHX chase assay". Data information: data are presented as mean±SD. ***P <0.001.

Figure S6

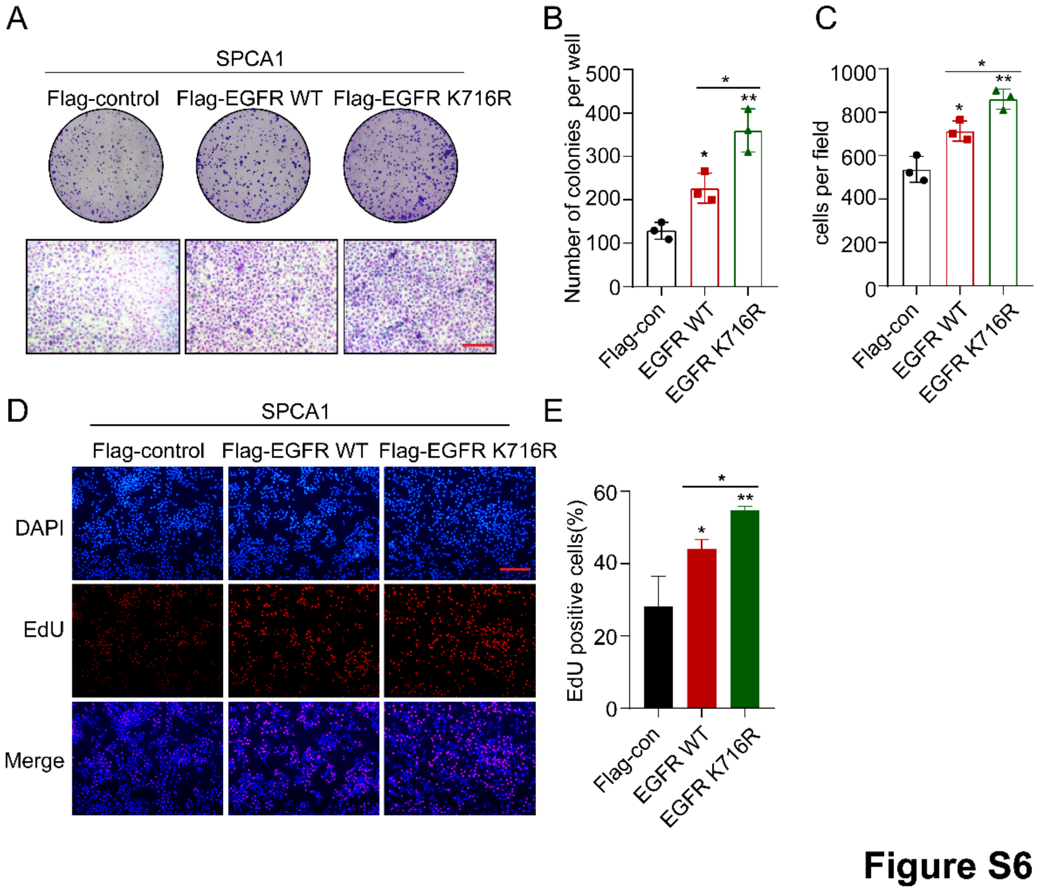

Supplemental Figure S6. Mutations of K716 in EGFR promotes NSCLC cell proliferation and migration

A, B, C The colony formation and migration of SPCA1 cells transfected with Flag-control, Flag-EGFR（WT）or Flag-EGFR（K716R）plasmids by colony formation assay and Transwell assay, n=3 independent experiments. Scale bars=50 μm. **D, E** Assessment of the proliferation of A549 cells transfected with Flag-control, Flag-EGFR (WT) or Flag-EGFR (K716R) plasmids by EDU assay visualized through EDU incorporation (red) and DAPI-stained nuclei (blue). Scale bars=50 μm. Data information: data are presented as mean±SD (n=3 independent experiments). **P* <0.05, ***P* <0.01.

Figure S7

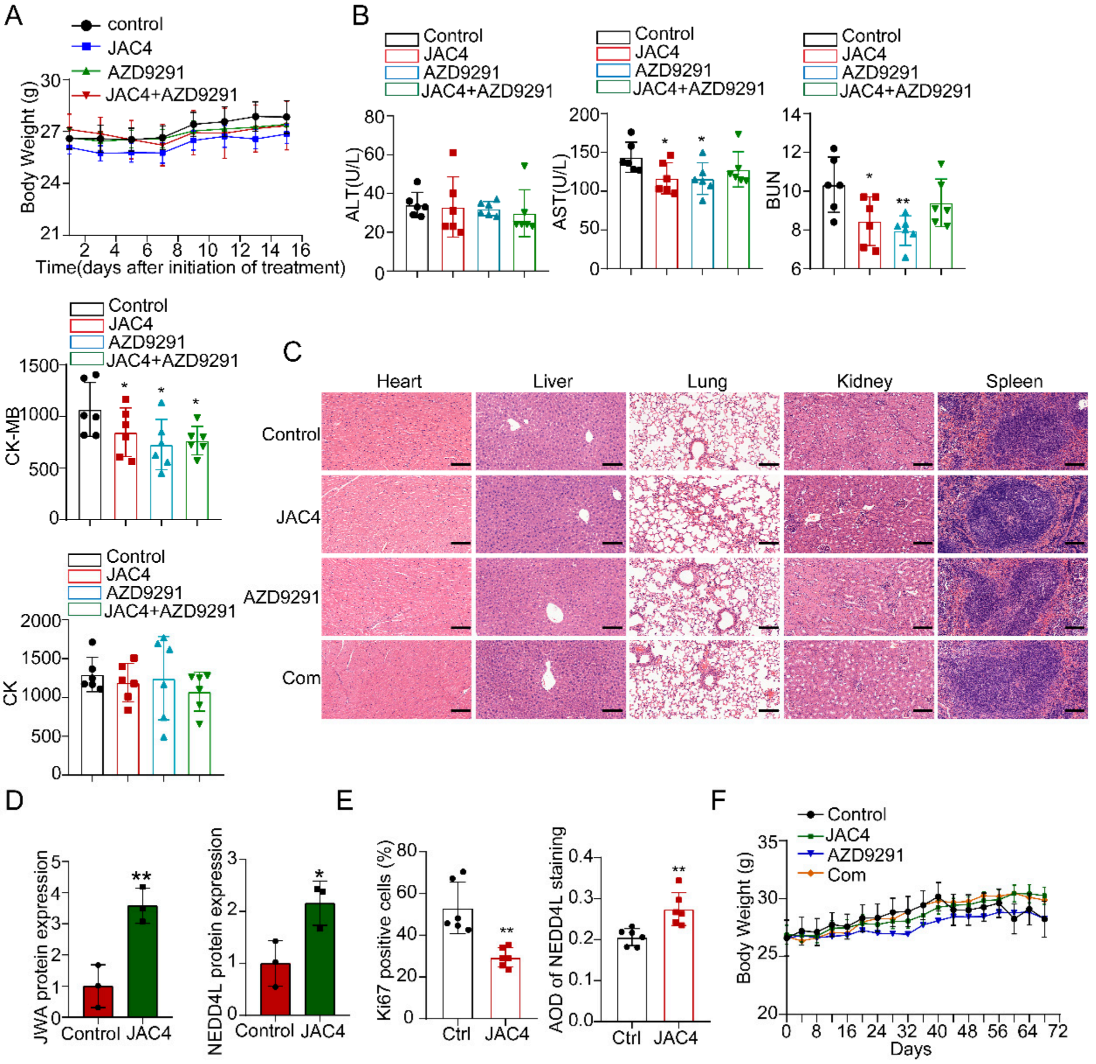

Figure S7

Supplemental Figure S7. Combination JAC4 and AZD9291 has no toxic side effect on the body

A Growth curves of body weight from different treatment groups in EGFR-mutant lung cancer cell tumor-bearing model (n=6). **B** Mouse serum biochemical indicators from different treatment groups in this model (n=6). **C** Histological evaluation of normal organs in mice. Scale bars=100 μm. **D** Analysis of relative JWA and protein levels via software Image J. **E** The quantification of Ki67 and NEDD4L staining was measured using Image J software, n=3 sections , 2 views per section. **F** Growth curves of body

weight from different treatment groups in EGFR-mutant lung cancer cell metastasis model (n=6). Data information: data are presented as mean±SD. *P <0.05, **P <0.01. Two-tail unpaired Student's t-test (D), one-way ANOVA test (B).

Figure S8

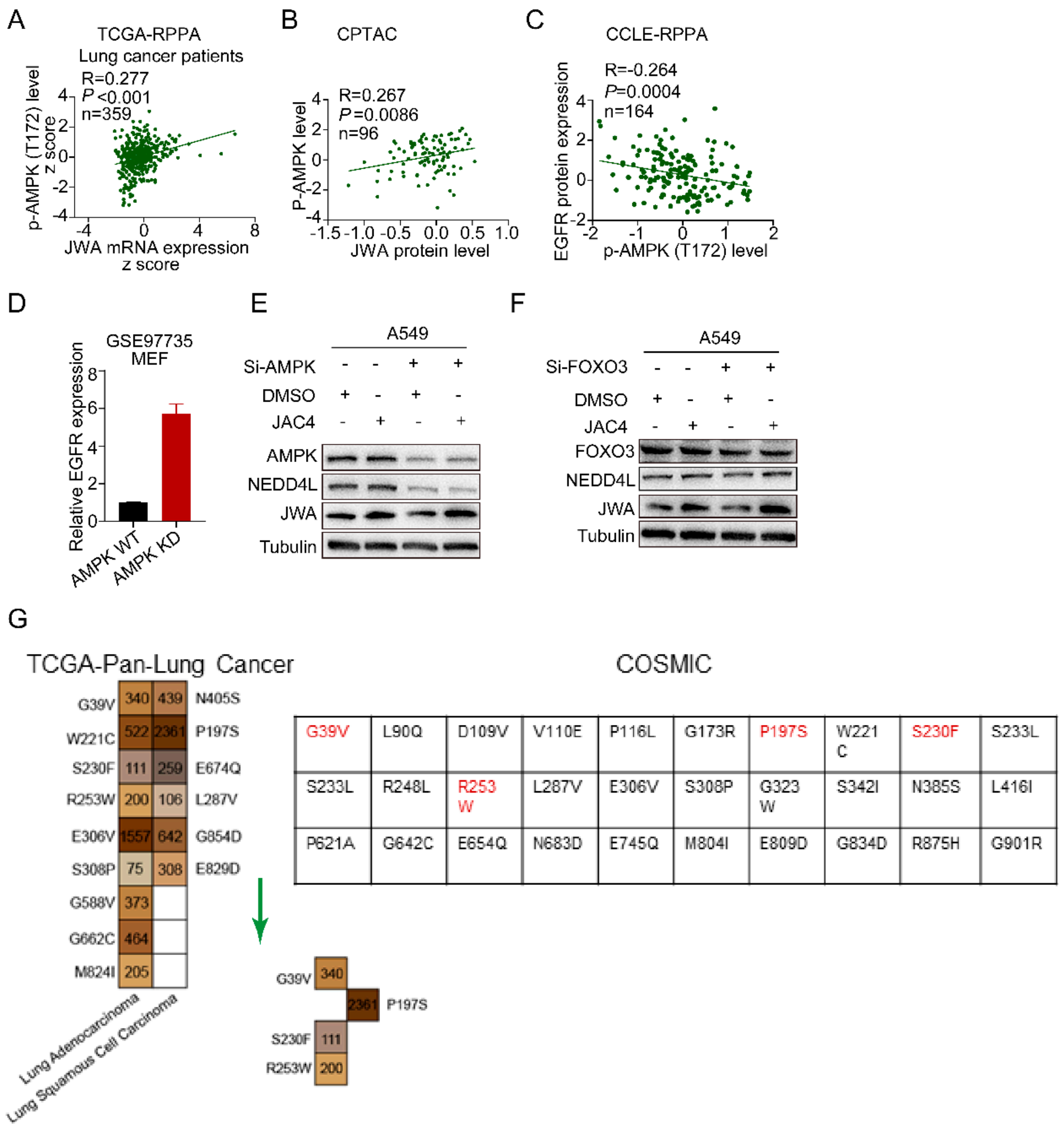

Figure S8

Supplemental Figure S8. Cancer-associated mutations NEDD4L regarding NSCLC

A, B Positive correlation between JWA expression and P-AMPK level based on analysis TCGA-RPPA and CPTAC-phosphoproteome. **C** Negative correlation between P-AMPK level and EGFR protein expression from analysis of CCLE-RPPA. **D** EGFR expression in RNA-seq between AMPK WT and AMPK KD MEF cells based on GSE97735. **E** IB analysis of A549 cells treated with 10 μM JAC4 for 24 h with or without

AMPK knockdown. **F** IB analysis of A549 cells treated with 10 μM JAC4 for 24 h with or without FOXO3 knockdown. **G** Cancer-associated mutations of NEDD4L in lung cancer tissues from COSMIC and TCGA data set. Data are presented as mean±SD. **P* <0.05, ***P* <0.01, ****P* <0.001. Pearson's correlation test (A-C).

Figure S9

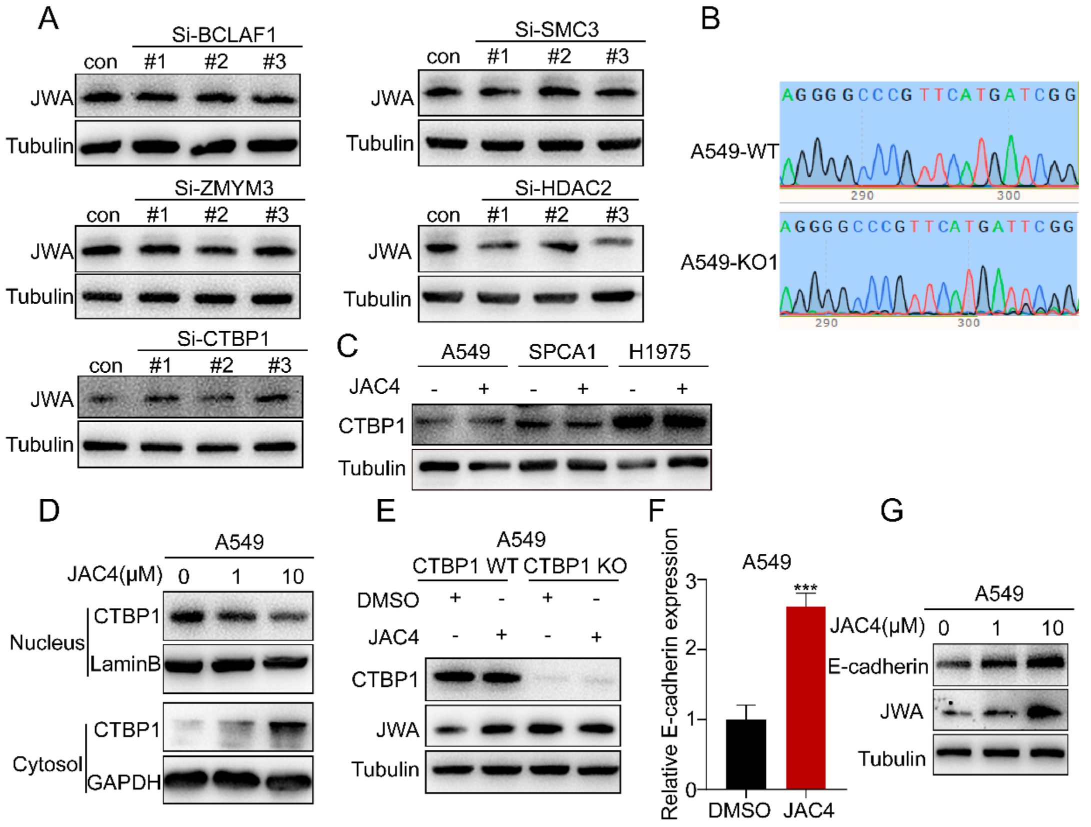

Figure S9

Supplemental Figure S9. Screening of transcriptional factors bound by JAC4

A IB analysis of JWA expression in HBE cells with or without several genes' knockdown. **B** CRISPR/Cas9-mediated knockout of CTBP1 in A549 cells using the Sanger sequencing. **C** IB analysis of CTBP1 protein expression in NSCLC cells (A549, SPCA1, NCI-H1975) treated with DMSO or 10 μM JAC4. **D** A549 cells were treated with indicated concentrations of JAC4 for 24 h, and IB analysis of CTBP1 protein were

detected in the nucleus and cytoplasm. **E** IB analysis of the protein expression of CTBPA and JWA in A549 cells with or without CTBP1 KO treated with DMSO or 10 μM JAC4. **F** mRNA expression of E-cadherin was determined by qRT-PCR. **G** IB analysis of the protein expression of E-cadherin in A549 cells treated with different concentrations of JAC4. Data are presented as mean±SD. ****P* <0.001.

Supplementary Tables

Supplementary Table S1: The sequences of siRNAs

siRNAs	Sequences (5'-3')
siBCLAF1#1	CAGUCUAGUUCUCGAUCAAGA
siBCLAF1#2	AGUUCUCGAUCAAGAUCAAGA
siBCLAF1#3	AGAUCAAGAUCUCAUUCUAGA
siSMC3#1	GGAUCUUAAUGAAGUCAAAGC
siSMC3#2	GCUUAGUGCUGAAAGACAAGA
siSMC3#3	GGAUAUUAGUAUAAUGAAAUG
siZMYM3#1	CGAUGUGUUCUACCUGCAACC
siZMYM3#2	GGUGCAAGACCCUGUGUAAGA
siZMYM3#3	CGCUCAAGUGUGUCACAAAGG
siHDAC2#1	GGUCAAUAAGACCAGAUAACA
siHDAC2#2	GGUCAGGUUUGGUAAACUAAG
siHDAC2#3	AGUUCAGUGUUAUAUGUAAUG
siCTBP1#1	GCUUCAACGUGCUCUUCUACG
siCTBP1#2	GCUCGCACUUGCUCAACAAGG
siCTBP1#3	CGUCAAGCAGAUGAGACAAGG
siFOXO3#1	GAGCTCTAGCTTCCCGTAT
siAMPK#1	GAGGAGAGCUAUUUGAUUATT

Supplementary Table S2: The primer sequences for qRT-PCR

Primer names	Sequences (5'-3')
GAPDH F	GTCTCCTCTGACTTCAACAGCG
GAPDH R	ACCACCCTGTTGCTGTAGCCAA
JWA F	GGTGGTCATGTTGGCGAGCTAT
JWA R	GGTTCCGAAGTCTCAACGATGC
EGFR F	AACTGTGAGGTGGTCCTTGG

EGFR R	GTTGAGGGCAATGAGGACAT
E-cadherin F	GCCTCCTGAAAAGAGAGTGGAAG
E-cadherin R	TGGCAGTGTCTCTCCAAATCCG

Supplementary Table S3: Antibodies for western blot and Co-IP

Antibody name	Source	Item number
AKT	Beyotime	AF0045
P-AKT（Ser473）	Beyotime	AF1546
P-AKT (Ser473)	CST	#4060
STAT3	Abcam	Ab68153
P-STAT3 (Tyr705)	CST	#9145
EGFR	CST	4267S
EGFR	Proteintech	18986-1-AP
PCNA	Proteintech	10205-2-AP
BAX	Proteintech	50599-2-AP
BCL2	Proteintech	12789-1-AP
NEDD4L	CST	4013S
NEDD4L	Proteintech	13690-1-AP
MIB1	Proteintech	11893-1-AP
WWP1	Proteintech	67804-1-Ig
SMURF1	Proteintech	55175-1-AP
RNF41	Proteintech	17233-1-AP
HA	Beyotime	AF5057
Flag	Beyotime	AF5051
AMPK	Beyotime	AF6195
P-AMPK (Thy172)	CST	2535T
Foxo3	Beyotime	AF609
UB	CST	3936T
UB	Santa	Sc-271289
Tubulin	Beyotime	AT819
CTBP1	Proteintech	10972-1-AP
HRP-labeled Goat mouse antibody	Beyotime	AF0216
HRP-labeled Goat rabbit antibody	Beyotime	AF0208
Flag	Sigma	F1804
GAPDH	Beyotime	AF0006
E-cadherin	Affinity	AF0131
Cleaved caspase 3	Proteintech	19677-1-AP
JWA	Self-made by the group	-
